# Digital literacy, psychological resilience, and work stress among nurses: the moderating role of ward environment

**DOI:** 10.3389/fpsyg.2026.1810798

**Published:** 2026-04-29

**Authors:** Yan Wu, Wanbin She, Qiyan Peng, Jile Li, Chenghong She

**Affiliations:** 1 Nursing & Health Sciences School, Leshan Vocational & Technical College, Leshan, China; 2 Faculty of Education Science, Leshan Normal University, Leshan, China

**Keywords:** digital literacy, moderating effect, nurses’ work stress, psychological resilience, ward environment

## Abstract

**Introduction:**

Nurses’ work stress remains a persistent global challenge, with the digital transformation of healthcare adding new complexities to their roles.

**Aim(s):**

This study aimed to explore the relationships among nurses’ digital literacy, psychological resilience, and work stress, and to investigate the moderating role of the ward environment in these mediation and moderation relationships.

**Design/methods:**

A cross-sectional study was conducted from June to July 2025 using anonymous online questionnaires completed by 302 active clinical nurses from Western China. Descriptive statistics, correlation analyses, and structural equation modeling (SEM) were performed. The PROCESS macro was employed to examine the hypothesized moderated mediation effects.

**Results:**

Nurses working in intensive care units (ICUs) and emergency rooms (ERs) reported significantly higher levels of work stress compared to those in general wards. The statistical indirect association between digital literacy and work stress through psychological resilience was fully mediated. A key finding was the significant moderating role of the ward environment. In the ER setting, digital literacy was directly associated with lower work stress (*β* = −0.185, *p* = 0.025) and indirectly associated with it through higher psychological resilience, with the combined variables accounting for 52.1% of the variance in stress. In contrast, the ICU environment was associated with a weaker negative relationship between psychological resilience and work stress (*β* for interaction = 0.293, *p* = 0.001).

**Conclusion:**

This study demonstrates that the ward environment plays a critical role in shaping how digital literacy and psychological resilience relate to nurses’ work stress. The findings suggest that to address work stress effectively, hospitals may consider implementing ward-specific strategies. Based on the observed associations, combining digital tool training with resilience-building initiatives could be beneficial in ERs. In ICUs, the findings point to the need for systemic interventions beyond individual-level resilience to address the high occupational demands.

## 1 Introduction

The World Health Organization (WHO, 2025) has recognized nurse work stress and burnout as a public health crisis with global, persistent, and systemic implications. A meta-analysis by Ilola et al. (2024) indicates a high prevalence of mental health disorders, such as burnout and anxiety, among nurses. The enduring nature of this crisis is underscored by data from the COVID-19 pandemic, which serves as a critical baseline illustrating the severity of the issue. For instance, during the pandemic, 36% of clinical nurses in Germany exhibited moderate or severe depressive symptoms (Boß et al., 2025), and in China, the prevalence of burnout and depressive symptoms among nurses was 48.2 and 64.1%, respectively (Zhang et al., 2024). These figures highlight a sustained mental health challenge that continues to pose a severe threat to healthcare quality and patient safety in the post-pandemic era. The widespread adoption of digital technologies, such as electronic health records and remote monitoring, has introduced new challenges, leading to a “digital overlay” of nursing work stress and potential overload. In this context, digital literacy and psychological resilience, as key individual resources, are considered crucial factors in mitigating nursing work stress.

Similarly, among ICU nurses in Nepal, the prevalence of depressive and anxiety symptoms reached 21.2 and 36.5%, respectively (Tamrakar et al., 2023). In the late stages of the pandemic in China, the prevalence of burnout and depressive symptoms among nurses was 48.2 and 64.1%, respectively, posing a severe threat to healthcare quality and patient safety. The widespread adoption of digital technologies, such as electronic health records and remote monitoring, has introduced new challenges, leading to a “digital overlay” of nursing work stress and potential overload. In this context, digital literacy and psychological resilience, as key individual resources, are considered crucial factors in mitigating nursing work stress.

## 2 Theoretical framework and hypothesis development

To investigate the complex relationships among digital literacy, psychological resilience, work stress, and the ward environment, this study is grounded in two complementary theoretical frameworks: the Conservation of Resources (COR) theory and the Job Demands-Resources (JD-R) model.

### 2.1 The conservation of resources (COR) theory and job demands-resources (JD-R) model as an integrative framework

COR theory (Hobfoll, 1989) posits that individuals strive to acquire, protect, and build resources—objects, personal characteristics, conditions, or energies that they value. Stress occurs when these resources are threatened, lost, or not regained after significant investment. In this framework, both digital literacy (a personal capability) and psychological resilience (a personal charact2-eristic) can be viewed as key resources that nurses can utilize to protect themselves against the resource-depleting demands of their work.

The JD-R model (Bakker and Demerouti, 2017) complements COR by suggesting that job demands and job resources are the two primary categories of working conditions. Job demands can lead to burnout and stress, while job resources can buffer this process and foster engagement. Crucially, the JD-R model posits that the impact of personal resources on work outcomes is contingent upon the specific job demands and resources present in the work environment. By integrating these theories, this study proposes that digital literacy (a resource) can foster psychological resilience (another resource), and together they can mitigate work stress (a negative outcome of resource loss). However, the strength of these relationships is expected to depend on the unique demands and resources characteristic of different ward environments (e.g., ER, ICU).

### 2.2 Hypothesis development

#### 2.2.1 The mediating role of psychological resilience (H1)

According to COR theory, resources do not exist in isolation but often travel in “caravans” (Hobfoll, 1989). This means that possessing one key resource can help individuals generate and protect other resources. In this study, digital literacy— the competence to effectively use digital tools and information — can be conceptualized as a foundational resource. Nurses with higher digital literacy may find it easier to access information, solve problems, and manage their workload. This competence can build a sense of mastery and control, which are core components of psychological resilience. Empirical evidence supports this link, demonstrating that digital competence is associated with positive psychological outcomes like self-efficacy and well-being (Li et al., 2023; Wu et al., 2021). Consequently, digital literacy is expected to be positively related to psychological resilience. Furthermore, psychological resilience is a well-established protective factor that helps individuals adapt to stress and adversity (Teng et al., 2021). A resilient nurse is better equipped to cope with the demands of the job, thereby reducing experienced work stress. We therefore hypothesize:

*H1*: Psychological resilience mediates the association between digital literacy and nurses' work stress.

#### 2.2.2 The moderating role of the emergency room environment (H2)

The JD-R model provides a lens for understanding how the work context can alter the relationship between personal resources and outcomes. The Emergency Room (ER) is characterized by high variability, unpredictability, time pressure, and critical decision-making. In such a high-demand environment, the instrumental value of digital.

literacy is likely amplified. Proficiency in using digital tools — such as electronic triage systems, rapid access to patient histories, and communication platforms — can directly reduce workload, minimize errors, and improve efficiency (Montazeri et al., 2021). This direct, instrumental benefit is a job resource that can directly counteract job demands. Therefore, while digital literacy may not directly relate to stress in lower-demand settings, its value as a direct coping tool becomes salient in the ER. Thus:

*H2*: The ER environment significantly moderates the direct relationship between digital literacy and work stress, such that the negative association is stronger in the ER than in non-ER settings.

#### 2.2.3 The moderating role of the intensive care unit environment (H3)

The Intensive Care Unit (ICU) presents a distinct context characterized by chronic, high-stakes demands, emotional intensity, and frequent exposure to patient death and suffering. This is a quintessential resource-depleting environment from a COR perspective. While psychological resilience is generally a protective resource, COR theory suggests that when resource loss is continuous and severe, the ability of any single personal resource to buffer stress may be overwhelmed. In the ICU, the relentless demands may mean that even highly resilient nurses experience high stress. This is not to say resilience is unimportant, but rather its protective relationship with stress may be significantly weakened or even nullified in the face of such extreme, systemic pressures. Empirical work has shown that the high-stress ICU environment can challenge the protective mechanisms of resilience (Mealer et al., 2012; Salluh et al., 2022). We therefore hypothesize:

*H3*: The ICU environment moderates the indirect relationship between digital literacy and work stress by attenuating the negative association between psychological resilience and work stress.

#### 2.2.4 The dual-pathway mechanism in the emergency room (H4)

**Figure 1:**
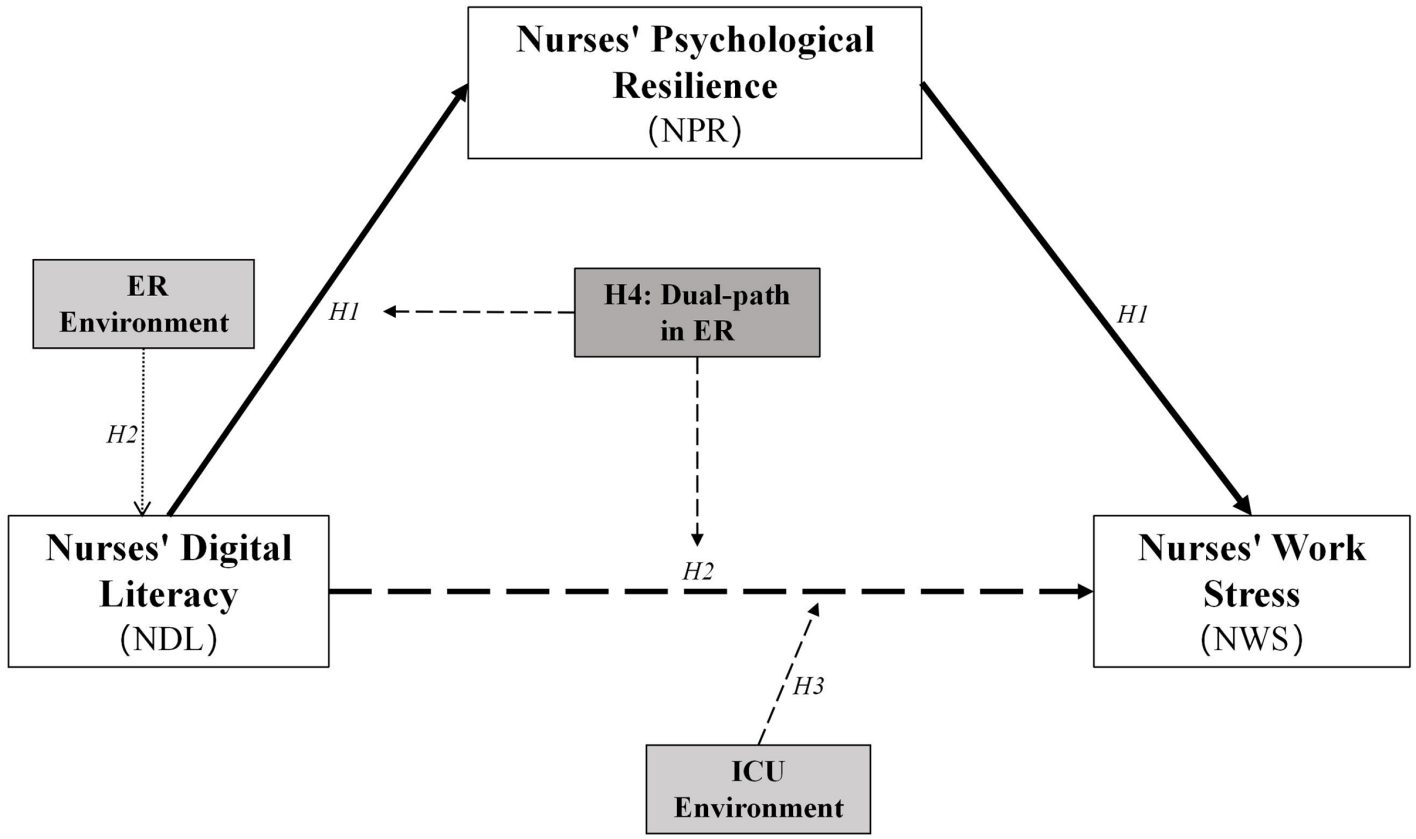
Conceptual model depicting the hypothesized relationships, including the mediating role of psychological resilience and the moderating roles of the ER and ICU environments.

Building on the theoretical arguments for H1 and H2, the ER context presents a unique case where both direct and indirect pathways may be active. As argued for H2, digital literacy in the ER serves as a direct, instrumental job resource that reduces stress by optimizing workflows. Simultaneously, as argued for H1, digital literacy serves as a personal resource that builds psychological resilience, which in turn helps nurses cope with stress. This suggests a dual pathway — one direct (technological) and one indirect (psychological) — through which digital literacy is associated with lower work stress specifically within the ER setting. This pattern represents a more complete integration of the JD-R model, where a personal resource (digital literacy) functions both as a direct job resource and as a builder of other personal resources (Figure 1). Thus:

*H4*: In the ER setting, digital literacy is associated with lower work stress through both direct and indirect (via psychological resilience) pathways, reflecting a dual-path mechanism.

## 3 Methods

This study employed a cross-sectional survey design. Data were collected anonymously online from nurses in 12 hospitals in Western China to balance convenience and data integrity.

### 3.1 Participants and sampling

The study participants were registered nurses actively working in clinical settings. Inclusion criteria consisted of having at least one year of work experience, engaging in clinical work for a minimum of 30 h per week, and providing informed consent. Nurses who were interns, undergoing advanced training, rehired after retirement, or on maternity leave, sick leave, or external training during the survey period were excluded.

The required sample size was calculated based on the requirements for Structural Equation Modeling (SEM) analysis. Following recommendations that the sample size should exceed 200 (Kumar and Natarajan, 2020; Reinartz et al., 2009) and be at least 10 times the number of questionnaire items (Ghanbar and Rezvani, 2023), a target sample of 280 was determined. Participants were recruited using a snowball sampling technique. Initially, 305 nurses were selected and invited to participate. All 305 questionnaires were distributed, and after removing invalid responses, 302 valid questionnaires were retained, yielding an effective response rate of 99.02%.

### 3.2 Data collection

Data collection took place between June and July 2025. The researchers developed an online questionnaire using the WPS Form mini-program within WeChat, which generated a QR code for distribution. The purpose and significance of the study were explained to potential participants. The questionnaire QR code was then distributed individually to nurses who expressed willingness to participate. The survey was designed to require respondents to answer all questions before submission.

### 3.3 Ethical considerations

This study was reviewed and approved by the Academic Ethics Committee of Leshan Vocational and Technical College (Approval No.: LVTCERC202502) on May 25, 2025. A written informed consent statement was prominently displayed on the first page of the questionnaire. It clearly stated that participation was voluntary and anonymous, and that participants could withdraw at any time without any negative consequences.

### 3.4 Instruments

*Hospital ward type (HWT)*: A general information questionnaire developed by the researchers collected data on gender, education level, age, hospital type, hospital level, hospital ownership, and ward type. Ward type was categorized into three groups: Emergency Room (ER), Intensive Care Unit (ICU), and General Ward (GW).

*Nurses’ work stress (NWS)* was assessed using the Nurses’ Work Stressors Scale compiled by Yu (2007). The 35-item scale assesses resilience levels across five dimensions: (a) professional role conflicts, (b) temporal workload distribution, (c) environmental resource adequacy, (d) complex patient management demands, and (e) administrative communication efficacy. Responses were recorded on a 4-point Likert scale from 1 (“strongly disagree”) to 4 (“strongly agree”), with higher scores indicating greater work stress. The scale demonstrated good reliability in the original study (Cronbach’s *α* = 0.94) (Yu, 2007) and in the current sample (Cronbach’s α = 0.911).

*Nurses’ digital literacy (NDL)* was measured using the Digital Literacy Evaluation Scale developed by Liu et al. (2025). This 21-item scale assesses digital literacy across five dimensions: (a) technological awareness, (b) information synthesis capacity, (c) virtual collaboration proficiency, (d) innovative application skills, and (e) cybersecurity stewardship. Responses were recorded on a 5-point Likert scale from 1 (“completely unimportant”) to 5 (“very important”), with higher scores reflecting more positive evaluations of digital literacy. The original study reported excellent reliability (Cronbach’s *α* = 0.941) (Liu et al., 2025), which was acceptable in this study (Cronbach’s α = 0.825).

*Nurses’ psychological resilience (NPR)* was evaluated using the Nurse Psychological Resilience Scale compiled by Lin et al. (2020). This 34-item scale assesses resilience levels across three dimensions: (a) contextual adaptation to clinical environments, (b) individual coping mechanisms, and (c) collaborative system rebuilding after adversity. Items are rated on a 5-point Likert scale from 1 (“strongly disagree”) to 5 (“strongly agree”), where higher scores denote higher levels of psychological resilience. The scale showed high reliability in its development (Cronbach’s *α* = 0.928) (Lin et al., 2020) and in the present study (Cronbach’s α = 0.909).

### 3.5 Data analysis

Statistical analyses were performed using IBM SPSS Statistics version 30.0. Descriptive statistics were computed based on participants’ demographic characteristics. Independent samples *t*-tests and one-way ANOVA were used to examine group differences. Pearson correlation analysis was conducted to explore the relationships among digital literacy, psychological resilience, and work stress across different ward environments.

To comprehensively test the theoretical framework, a complementary methodological approach was adopted. The rationale for this dual approach is that SEM and PROCESS serve distinct but complementary purposes.

*Structural equation modeling (SEM)*: SEM was first employed as a confirmatory tool to provide a holistic test of the overall theoretical model. Using IBM SPSS AMOS version 30.0, we assessed the fit of the latent variable model and the structural relationships among all variables simultaneously. This provided a macro-level validation of our conceptual framework, including the convergent and discriminant validity of our multi-dimensional constructs.

*PROCESS macro for hypothesis testing*: Subsequently, the PROCESS macro (Hayes, 2013) version 4.3 in SPSS was used as a focused, hypothesis-testing tool. This allowed for a precise examination of specific conditional indirect effects and interactions with dummy-coded moderator variables (Dept_ER, Dept_ICU), which is essential for testing the nuanced relationships hypothesized in H2, H3, and H4. Using PROCESS for these specific tests avoids the complexity of latent interaction modeling in SEM and provides easily interpretable output for our distinct moderators. A stepwise approach was employed:

- *Model 4* was used to test the core mediation effect of psychological resilience (H1).
- *Model 1* was used to test the simple moderation of the direct path by the ER environment (H2).
- *Model 58* was used to test the moderated mediation effect of the ICU environment, focusing on the moderation of the b-path (NPR -> NWS) (H3).
- *Model 7* was used to examine the conditional indirect effect (a-path) in the ER context (H4).

All PROCESS analyses were performed with 10,000 bootstrap samples to generate 95% confidence intervals.

### 3.6 Data bias verification and reliability of statistical analysis

#### 3.6.1 Data preprocessing and quality control

To address scaling differences between the work stress scale (4-point Likert) and the psychological resilience/digital literacy scales (5-point Likert), all variable scores were converted to Z-scores to eliminate scale unit effects (Collins et al., 2015). Given minor deviations from normality, a Winsorization procedure (1% bilateral) was applied to control the influence of extreme values, a standard practice to enhance the robustness of parametric analyses (Collins et al., 2015). This adjustment improved the distributional properties of the data: for digital literacy, kurtosis improved from −0.24 to −0.878 and the standard deviation increased from 0.50 to 0.67; for work stress, skewness improved from −0.09 to −0.14 (standard deviation changed from 0.63 to 0.71). The absolute values of skewness for all variables were less than 0.44 (Standard Error = 0.14), and the absolute values of kurtosis were less than 0.88 (Standard Error = 0.28). These values met the criterion of |skewness/kurtosis| < 2 for approximating a normal distribution (Streiner et al., 2024).

#### 3.6.2 Model fit assessment and reliability verification

The hypothesized structural equation model (SEM) was constructed using AMOS 30.0 software and tested via the maximum likelihood estimation method. The model fit indices, presented in Table 1, indicate a good fit to the data: χ^2^/df = 1.973 (< 3), RMSEA = 0.057 (90% CI [0.044, 0.070]), and GFI = 0.940 (> 0.90). The comparative fit indices (CFI = 0.977, NFI = 0.954, IFI = 0.977), all exceeding 0.95, indicate that the model fits significantly better than the independent model. The parsimony fit indices (PNFI = 0.709, PCFI = 0.726), both greater than 0.50, reflect good model parsimony. Composite reliabilities for NDL, NPR, and NWS were 0.837 0.825, and 0.8, respectively; average variances extracted were 0.57, 0.616, and 0.504, all exceeding the 0.50 threshold, supporting convergent validity. Collectively, these indices meet the standards for good model fit (Kline, 2018), suggesting the model has acceptable stability and explanatory power and is suitable for hypothesis testing and subsequent path analysis.

**Table 1:** Structural equation model fit indices.

Fit indices	Evaluation criteria	Model values	Goodness-of-Fit assessment
χ^2^/df	<3	1.973	Good
RMSEA	<0.08	0.057 (90%CI:0.044–0.070)	Acceptable
GFI	>0.90	0.940	Good
CFI	>0.95	0.977	Excellent
NFI	>0.90	0.954	Good
IFI	>0.95	0.977	Excellent
TLI	>0.95	0.969	Good
PNFI	>0.50	0.709	Good
PCFI	>0.50	0.726	Good
AIC	< Saturated Model	237.922 (Saturated: 240.000)	Excellent
Construct	CR	AVE	
NDL	0.837	0.570	
NPR	0.825	0.616	
NWS	0.800	0.504	

Reference standards based on Hu and Bentler (1999) and Kline (2018).

A multiple linear regression model was established with standardized work stress (Z-score) as the dependent variable and standardized psychological resilience, digital literacy (Z-scores), and department type as predictor variables. The effective sample size (*N* = 302) satisfied the requirement of > 50 + 8 k (where k = 3 predictors). Statistical power exceeded 99% (for detecting R^2^ = 0.29, *α* = 0.05). The model was significant, *F* (3, 298) = 41.464, *p* < 0.001, with R^2^ = 0.294 and adjusted R^2^ = 0.287, indicating that the predictor variables jointly explained 28.7% of the variance in work stress.

#### 3.6.3 Multicollinearity and residual diagnostics

**Figure 2:**
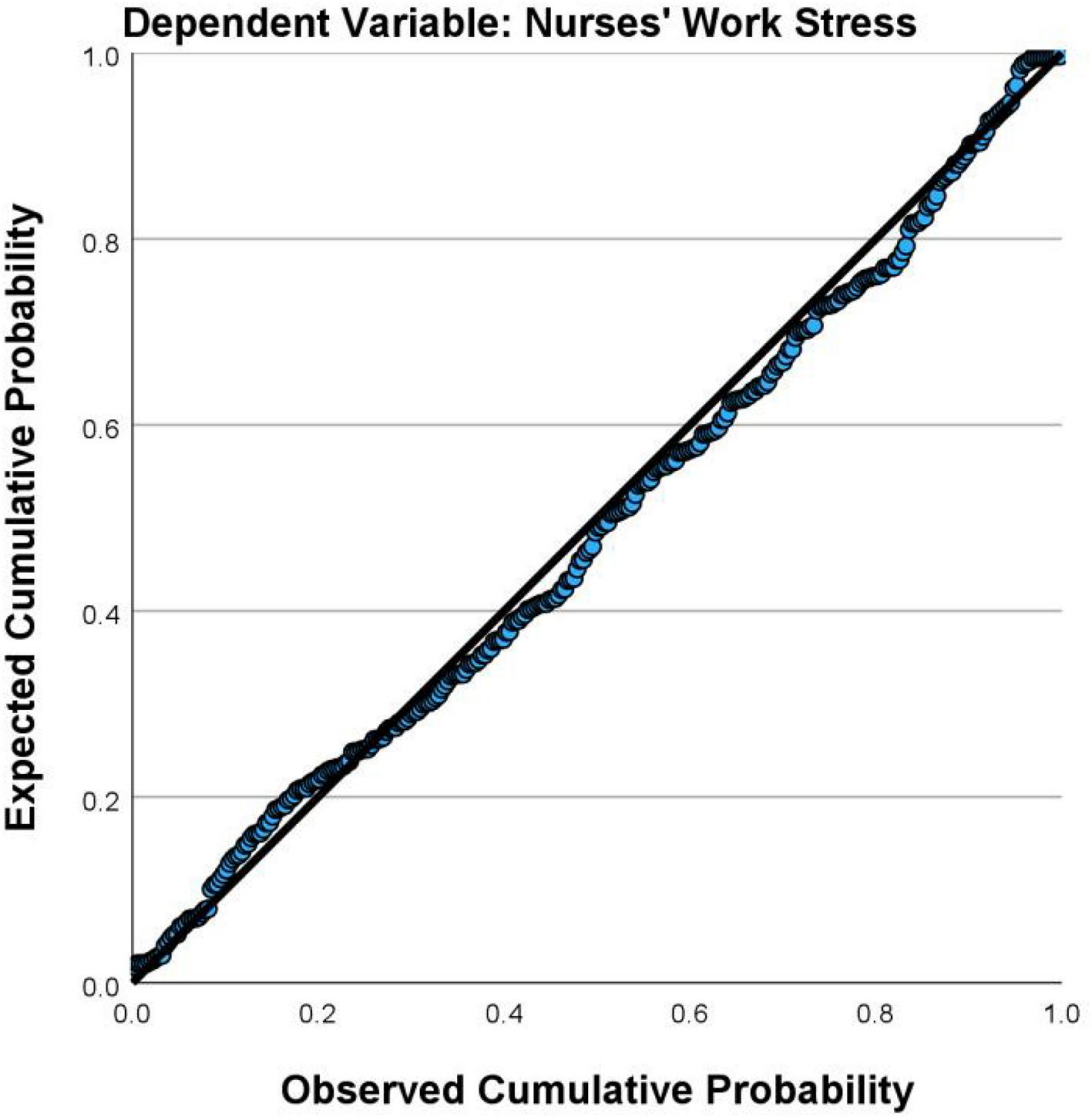
Normal P–P plot of regression standardized residuals.

The Variance Inflation Factor (VIF) for all variables was below 1.32 (Department Type = 1.14, Digital Literacy = 1.24, Psychological Resilience = 1.32), well under the critical threshold of 5. The maximum condition index was 5.37 (< 30), and no single dimension simultaneously explained a high proportion of variance for multiple variables (e.g., Department Type accounted for 97% of the variance in Dimension 4), confirming no threat of multicollinearity. The standardized residuals had a mean of 0 (SD = 0.995), with 95% of residuals falling within [−1.95, 1.95]. Three cases had absolute standardized residuals exceeding 2.5 (max |SR| = 2.705). However, Cook’s distance values (max D = 0.12 < 1) confirmed no influential cases. The scatterplot of residuals against predicted values showed a random distribution, with no observable pattern of heteroscedasticity (Figure 2).

#### 3.6.4 Common method bias assessment

Given the cross-sectional, self-reported design, Harman’s single-factor test was conducted to check for common method variance. An exploratory factor analysis of all scale items revealed that the first factor accounted for 37.7% of the total variance, which is below the 40% threshold, suggesting that common method bias was not a severe threat in this study (Podsakoff et al., 2003).

To further assess this, a full collinearity test was conducted, as recommended by Kock (2015). All variance inflation factors (VIFs) derived from a regression model including all latent variables were below the conservative threshold of 3.3 (VIF range = 1.02–1.32). This provides additional evidence that common method bias is unlikely to be a significant threat to the validity of our findings.

### 3.7 Operationalization of ward environment for statistical analyses

To provide a coherent multi-level analysis of the ward environment as a single underlying construct, we operationalized it as a contextual risk level in two complementary ways to address different analytical objectives, as summarized in Table 2. For the SEM analysis, we used a dichotomous variable (HWT) to compare high-acuity wards (ICU and ER combined) against general wards, providing a parsimonious test of the overall structural model. For the PROCESS analyses, we used context-specific dummy variables (Dept_ER, Dept_ICU) to allow for a granular examination of the unique moderating patterns hypothesized for each specific setting.

**Table 2:** Variable coding forward environment.

Analysis	Variable name	Coding scheme	Interpretation of beta
SEM	HWT (Hospital Ward Type)	1 = High-Risk Ward (ICU & ER combined); 0 = General Ward	A negative coefficient indicates higher stress in high-risk wards (due to the coding).
PROCESS	Dept_ER	1 = Emergency Room; 0 = Non-ER	Direct interpretation: a positive coefficient means ER nurses have higher stress.
PROCESS	Dept_ICU	1 = Intensive Care Unit; 0 = Non-ICU	Direct interpretation: a positive coefficient means ICU nurses have higher stress.

The SEM analysis used the combined HWT variable to create a parsimonious structural model comparing high-acuity to general wards. The PROCESS analyses used the disaggregated dummy variables (Dept_ER, Dept_ICU) to allow for a more nuanced exploration of the distinct moderating patterns hypothesized for the ER and ICU environments.

## 4 Results

### 4.1 Descriptive analysis of variables

The majority of participants were female (96.69%), and the largest age group was 31–40 years old (44.04%). As shown in Table 3, digital literacy (NDL) scores differed significantly based on age and ward type. Nurses aged 31–40 and those working in general wards reported significantly higher levels of digital literacy (*F* = 6.079, *p* < 0.01; *F* = 9.036, *p* < 0.001, respectively). Both work stress (NWS) and psychological resilience (NPR) showed significant differences according to ward type. Nurses in the ICU reported the highest levels of work stress (*F* = 126.213, *p* < 0.001) and also higher psychological resilience (*F* = 31.029, *p* < 0.001) compared to other wards.

**Table 3:** Differences in digital literacy, work stress, and psychological resilience across demographic characteristics (*N* = 302).

Characteristics	Categories	*N* (%)	NDL		NWS		NPR	
			Mean ± SD	*t* or *F*	Mean ± SD	*t* or *F*	Mean ± SD	*t* or *F*
Age (years)	≤30	94(31.13)	4.24 ± 0.48	6.079**	2.55 ± 0.66	2.837	3.97 ± 0.63	2.655
	31–40	133(44.04)	4.40 ± 0.48		2.71 ± 0.65		4.04 ± 0.56	
	≥41	75(24.83)	4.18 ± 0.52		2.53 ± 0.55		3.84 ± 0.52	
Gender	Male	10(3.31)	4.32 ± 0.45	−0.153	2.83 ± 0.69	−1.118	4.05 ± 0.61	−0.474
	Female	292(96.69)	4.30 ± 0.50		2.61 ± 0.63		3.97 ± 0.58	
Hospital Ward type	ER	112(37.09)	4.18 ± 0.46	9.036***	2.83 ± 0.43	126.213***	3.66 ± 0.43	31.029***
	ICU	110(36.42)	4.28 ± 0.52		2.91 ± 0.53		4.18 ± 0.58	
	GW	80(26.49)	4.48 ± 0.47		1.90 ± 0.42		4.11 ± 0.57	

ER, emergency room; ICU, intensive care unit; GW, general ward; ***p < 0.001; **p < 0.01; *p < 0.05.

### 4.2 Structural relationships and direct effects among variables

#### 4.2.1 Operational definitions of variables and analytical approach

This study employed a multi-tiered operationalization scheme to examine the associations involving the ward environment. In the structural equation modeling (SEM) analysis, ward type was operationalized as a dichotomous variable (HWT), coded as 1 for high-risk wards (including ICU and Emergency Department) and 0 for general wards. For the PROCESS model analysis, to further explore distinct patterns of associations across different ward environments, ward type was disaggregated into two independent dichotomous variables: Dept_ER (Emergency Room = 1, non-Emergency Room = 0) and Dept_ICU (ICU = 1, non-ICU = 0). This complementary analytical strategy was designed to investigate how the ward environment relates to the study variables from both overarching and setting-specific perspectives. Although this approach increases analytical complexity, it facilitates a more comprehensive understanding of the interrelationships. All analyses clearly specified variable definitions and coding schemes to ensure transparency and replicability of the findings.

#### 4.2.2 Analysis of inter-variable correlations

Table 4 presents the results of the correlation analysis among the study variables. For the dichotomous variable — hospital ward type (HWT) — its associations with continuous variables were examined using the point - biserial correlation coefficient (r_pb). The analysis revealed a significant positive correlation between ward type and work stress (r_pb = 0.668, *p* < 0.001), indicating that nurses working in high- risk wards tended to report higher levels of work stress. Psychological resilience was negatively correlated with work stress (*r* = −0.214, *p* < 0.001) and positively correlated with ward type (r_pb = 0.407, *p* < 0.001). Digital literacy showed a significant negative correlation with work stress (*r* = − 0.201, *p* < 0.001) and a significant positive correlation with ward type (r_pb = 0.236, *p* < 0.001). In addition, a significant positive correlation was observed between digital literacy and psychological resilience (*r* = 0.431, *p* < 0.001).

**Table 4:** Correlations among key variables (*N* = 302).

Variable	1	2	3	4
NDL	1			
NPR	0.431***	1		
NWS	−0.201***	−0.214***	1	
HWT	0.236***	0.407***	0.668***	1

***p < 0.001; NDL, digital literacy; NPR, psychological resilience; NWS, work stress; HWT, hospital ward type (0 = General Ward, 1 = High-Risk Ward). Point-biserial correlation was used for analyses involving HWT.

#### 4.2.3 Structural equation modeling path analysis

Table 5 presents the standardized path coefficient estimates from the structural equation model. Consistent with the coding scheme described in section 3.7 (where HWT = 1 for high-risk wards), the negative path coefficient from Hospital Ward Type (HWT) to Work Stress (NWS) (*β* = −0.488, *p* = 0.006) indicates that work stress is significantly higher in high-risk wards (ICU and ER combined) compared to general wards.

**Table 5:** Standardized estimates for paths and covariances in the SEM.

Path/Covariance	Β/Cov	S. E.	C. R.	*p*	Sig.
NWS<--- NPR	−0.117	0.094	−1.250	0.211	ns
NWS<--- NDL	0.065	0.125	0.523	0.601	ns
NWS<--- HWT	−0.488	0.178	−2.740	0.006	**
NDL<-->HWT	0.015	0.006	2.483	0.013	*
NPR<-->HWT	0.077	0.017	4.465	<0.001	***
NDL<-->NPR	0.061	0.015	4.223	<0.001	***

All estimates are standardized; ***p < 0.001; **p < 0.01; *p < 0.05; ns, nonsignificant; NWS, work stress; NPR, psychological resilience; NDL, digital literacy; HWT, hospital ward type.

The direct association between Psychological Resilience (NPR) and Work Stress (NWS) was not statistically significant (*β* = −0.117, *p* = 0.211). Similarly, the direct association between Digital Literacy (NDL) and Work Stress was also non-significant (*β* = 0.065, *p* = 0.601). These findings suggest the need to further examine potential indirect pathways through which psychological resilience and digital literacy may be related to work stress.

**Figure 3:**
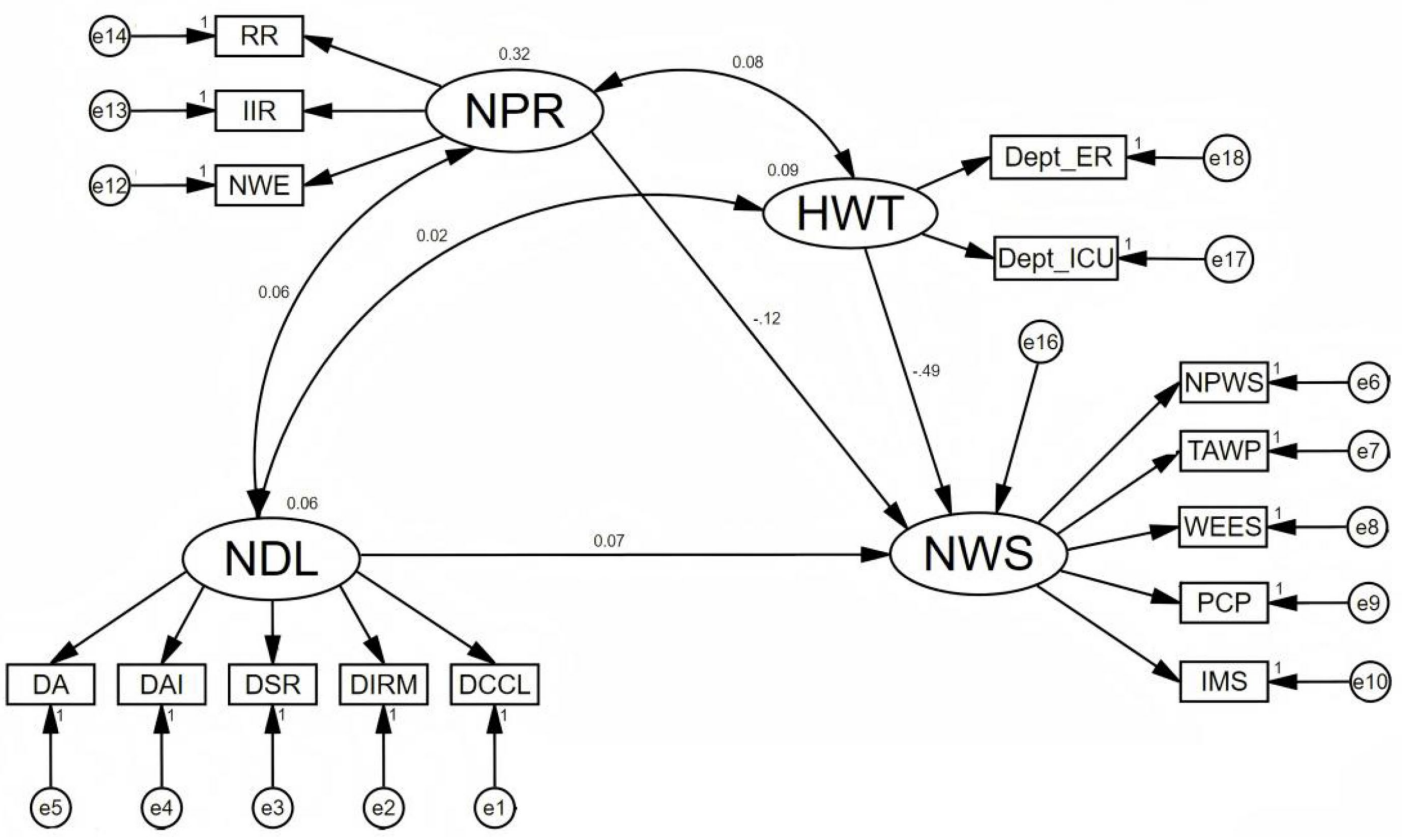
Structural equation model of variables.

Covariance analysis revealed significant positive relationships between Hospital Ward Type and Psychological Resilience (Cov = 0.077, *p* < 0.001), between Hospital Ward Type and Digital Literacy (Cov = 0.015, *p* = 0.013), and between Digital Literacy and Psychological Resilience (Cov = 0.061, *p* < 0.001), as illustrated in Figure 3.

### 4.3 Mediation and moderation effects

Data were analyzed using Hayes’ PROCESS macro to examine the relationships among digital literacy, psychological resilience, the ward environment, and nurses’ work stress. The results are as follows:

#### 4.3.1 Mediation effect of psychological resilience

PROCESS Model 4 was employed to examine the mediating role of psychological resilience in the relationship between digital literacy and work stress. The results (see Table 6) indicated that digital literacy was significantly positively associated with psychological resilience (*β* = 0.420, *p* < 0.001). After accounting for digital literacy, psychological resilience was significantly negatively associated with work stress (*β* = −0.152, *p* = 0.002). Bootstrap analysis with 10,000 resamples revealed that the indirect association between digital literacy and work stress through psychological resilience was.

**Table 6:** Decomposition of the mediation effect through psychological resilience.

Path	*β*	SE	*t*	*p*	95% CI/BootCI
NDL → NPR	0.420	0.054	7.741	<0.001	[0.313, 0.527]
NPR → NWS	−0.152	0.049	−3.111	0.002	[−0.249, −0.056]
Indirect Effect	−0.064	0.022	-	-	[−0.109, −0.022]*
Direct Effect	−0.005	0.050	−0.098	0.922	[−0.104, 0.094]

Based on 10,000 bootstrap samples; effects are significant if 95% CI excludes zero. Model fit: NPR model: R^2^ = 0.306, *F*(3, 298) = 43.69, *p* < 0.001; NWS model: R^2^ = 0.467, *F*(4, 297) = 65.08, *p* < 0.001.

−0.064, with a 95% confidence interval (CI) of [−0.109, −0.022]. Since the 95% CI did not include zero, this indirect association was statistically significant. The direct association between digital literacy and work stress was not significant (*β* = −0.005, *p* = 0.922). In summary, the relationship between digital literacy and work stress was fully mediated by psychological resilience, thereby supporting research Hypothesis H1.

#### 4.3.2 Moderating effect of the ward environment

To examine the moderating role of the ward environment, analyses were conducted using Model 1, Model 7, and Model 58, respectively. The results are presented in Table 7.

**Table 7:** Test results for the moderating effect of department type.

Path	Condition	*β*	SE	*t*	*p*	95% CI
NDL → NWS	Non-ER	−0.015	0.056	−0.271	0.786	[−0.125, 0.095]
NDL → NWS	ER	−0.185	0.083	−2.246	0.025*	[−0.348, −0.023]
Dept_ER → NWS	-	0.998	0.079	12.639	<0.001	[0.842, 1.153]*
NDL × Dept_ER	-	−0.170	0.100	−1.706	0.089	[−0.367, 0.026]
Conditional Direct	Non-ER	−0.015	0.056	−0.271	0.787	[−0.125, 0.095]
Effect	ER	−0.185	0.083	−2.246	0.025	[−0.348, −0.023]
Conditional	Non-ER	−0.117	0.031			[−0.182, −0.062]*
Indirect Effect	ER	−0.131	0.033			[−0.198, −0.068]*
Moderation Index		−0.014	0.029			[−0.072, 0.045]
NPR → NWS	Non-ICU	−0.274***	0.061	−4.513	<0.001	[−0.394, −0.155]
NPR → NWS	ICU	0.018	0.071	0.259	0.796	[−0.121, 0.158]
Dept_ICU → NWS	-	1.094	0.078	13.979	<0.001***	[0.940, 1.248]
NPR × Dept_ICU	-	0.293	0.089	3.290	0.001	[0.118, 0.468]
Conditional Direct	Non-ICU	−0.1209	0.0614	−1.969	0.050	[−0.2418, 0.0000]
Effect	ICU	0.0007	-	-	-	[−0.1202, 0.1216]
Conditional	Non-ICU	−0.098	0.028			[−0.160, −0.050]*
Indirect Effect	ICU	0.009	0.048			[−0.078, 0.110]
Moderation Index		0.108	0.055			[0.007, 0.224]*

*p < 0.05; **p < 0.01; ***p < 0.001. Model Fit: NPR Model: R^2^ = 0.309, *F*(4,297) = 33.27, *p* < 0.001; NWS Model: R^2^ = 0.486, *F*(5,296) = 55.95, *p* < 0.001; ND Model: R^2^ = 0.455, *F*(4, 297) = 62.00, *p* < 0.001. BootCI based on 10,000 samples.

After controlling for the ICU environment, the association between digital literacy and work stress was marginally moderated by the Emergency Room (ER) environment (*β* = −0.170, *p* = 0.089). Simple slope analysis indicated that within the ER environment setting, digital literacy was significantly negatively associated with work stress (*β* = −0.185, *p* = 0.025). In contrast, this association was not significant in the non-ER environment (*β* = −0.015, *p* = 0.787). This result supports Hypothesis H2.

Although the indirect association between digital literacy and work stress through psychological resilience was significant in both non-ER (*β* = −0.117, 95% CI [−0.182, −0.062]) and ER (*β* = −0.131, 95% CI [−0.198, −0.068]) settings, the index of moderated mediation was not statistically significant (as the 95% CI included zero). This suggests that the ER environment only marginally moderates the mediating role of psychological resilience in the relationship between digital literacy and work stress (see Table 6).

After controlling for the ER environment, although the association between digital literacy and work stress was not moderated by the ICU environment (95% CI included zero), the relationship between psychological resilience and work stress was significantly moderated by the ICU environment (*β* = 0.293, *p* = 0.001). Simple slope analysis revealed that in the non-ICU environment, psychological resilience had a significant negative association with work stress (*β* = −0.274, *p* < 0.001). However, this association was no longer significant in the ICU environment (*β* = 0.018, *p* = 0.796). The index of moderated mediation (Index = 0.108) was significant, with a 95% Bootstrap confidence interval of [0.007, 0.224] excluding zero. This indicates that the ICU environment was associated with a weaker negative relationship between psychological resilience and work stress (see Table 6), supporting Hypothesis H3.

Furthermore, one-way ANOVA indicated significant differences in work stress levels among nurses from different wards. Work stress levels were significantly higher for both ICU nurses (*β* = 1.094, *p* < 0.001) and ER nurses (*β* = 0.998, *p* < 0.001) compared to nurses in general wards (see Table 6).

#### 4.3.3 Integrated model of moderated mediation in the emergency room

Integrating the analytical results from Model 1, Model 7, and Model 58 (see Table 6), the pathways within the ER environment were examined. The results indicated that for nurses in the ER, digital literacy was associated with work stress through two significant pathways: (1) a direct negative association (*β* = −0.185, *p* = 0.025), and (2) an indirect pathway through psychological resilience (*β* = −0.131, 95% Boot CI [−0.200, −0.071]). The total association between digital literacy and work stress was −0.316. This suggests that within the ER, higher levels of digital literacy were associated with lower work stress. This integrated model collectively accounted for 52.1% of the variance in work stress among ER nurses. In summary, for ER nurses, digital literacy is not only directly negatively associated with work stress but is also related to it through the mediating role of psychological resilience. These findings support Hypothesis H4.

## 5 Discussion

Guided by the Conservation of Resources (COR) theory and the Job Demands-Resources (JD-R) model, this study examined the complex mechanisms through which digital literacy, psychological resilience, and the ward environment influence work stress among nurses. The main findings confirm the hypothesized model, revealing significant mediating and moderating effects among the variables.

### 5.1 The mediating role of psychological resilience

A central finding of this study is the full mediating role of psychological resilience in the relationship between digital literacy and work stress. Digital literacy was not directly associated with work stress but was indirectly associated with lower stress through its positive relationship with psychological resilience. This finding aligns with the “technological competence-psychological resource” transformation framework proposed by Fornés-Vives et al. (2016), which suggests that digital competencies may be linked to occupational well-being through their relationship with psychological capital. This result is also consistent with the work of Ou Yang (2020), who found that nurses’ digital competence is related to work adaptation through the mediating role of self-efficacy, a construct closely associated with psychological resilience. Furthermore, our findings correspond with the conclusions of Bock et al. (2025) and Li et al. (2023), indicating that digital training is associated with measurable emotional relief and higher work-related well-being, even over short timeframes. In summary, this study highlights that psychological resilience serves as a key mechanism through which digital literacy is related to lower work stress in the nursing context. These findings suggest that combining psychological resilience interventions with digital technology training may represent a practical approach to addressing stress among nurses. However, due to the inherent limitations of the cross-sectional design, the directional nature of the relationships among variables (e.g., whether digital literacy is associated with resilience or vice versa) and the temporal sequence of the mediation process (X → M → Y) require further verification through future longitudinal or experimental studies.

### 5.2 The contextual imperative: moderating role of ward environment

A key contribution of this research is its identification of the ward environment as an important contextual factor significantly moderating the relationships among digital literacy, psychological resilience, and work stress. The study found that the ward environment itself was independently and substantially associated with work stress. Notably, the ICU environment was associated with higher stress levels (ICU nurses reported stress levels 1.094 units higher than non-ICU nurses), highlighting the strong relationship between specific workplace demands and the stress experience.

The results further indicate that the relationship between digital literacy and work stress is closely tied to departmental characteristics. In the Emergency Room (ER) setting, digital literacy was directly and negatively associated with work stress (*β* = −0.185). This suggests that in a high-paced, high-decision-pressure environment like the ER, proficiency with digital tools (e.g., electronic triage systems) may be related to lower time pressure and decision-making burden through optimized workflows and information access. This finding is consistent with observations in the ER by Montazeri et al. (2021) and aligns with the perspectives of McGonigle and Mastrian (2022). Conversely, in non-emergency departments, no direct association between digital literacy and work stress was observed, implying that its relevance may be realized primarily through other indirect pathways, such as its positive relationship with psychological resilience. This disparity underscores the critical role of the ward environment in shaping whether the “instrumental value” of digital technology is evident. Our findings support the view of Xu and Zhao (2024) that organizational contexts are associated with the extent to which individual resources relate to workplace outcomes.

### 5.3 The attenuation of resilience in high-stakes environments

A particularly noteworthy finding is that the high-intensity, high-complexity ICU environment was associated with a weaker negative relationship between psychological resilience and work stress. While psychological resilience was significantly negatively associated with work stress in non-ICU departments (*β* = −0.274), this association was not observed within the ICU setting (*β* = 0.018, n.s.).

This pattern aligns with the core tenet of COR theory (Hobfoll, 1989): when individuals experience continual resource depletion (as may occur in the ICU environment), existing personal resources (such as psychological resilience) may show weaker associations with outcomes or may not demonstrate their typical protective relationships. This finding does not imply that resilience is unimportant for ICU nurses; rather, it suggests that its protective associations may be overwhelmed by the extreme and sustained job demands. The higher mean resilience scores observed in ICU nurses (Table 2) may reflect a “contextual resilience” developed through repeated exposure to critical situations (Abbasalizadeh et al., 2024; Mealer et al., 2012), rather than a trait that fully buffers stress. Our findings are consistent with the cautions raised by Mealer et al. (2012), the results of Du (2019), and the meta-analytic conclusions of Kunzler et al. (2022) and Salluh et al. (2022). This finding suggests that for ICU nurses, individual-level resilience may show limited association with work stress in the context of extreme job demands. Systemic interventions—such as workflow optimization and structured psychological support—may be necessary to support their resource protection mechanisms.

### 5.4 The dual-pathway mechanism in the emergency department

Our findings indicate that within the emergency department setting, digital literacy is not only directly negatively associated with work stress but is also related to it through its positive association with psychological resilience. This pattern suggests that digital literacy may function as a contextually relevant resource, showing relationships with work stress through both direct (technology-enabled) and indirect (psychology-empowered) pathways. This dual-pathway pattern aligns with the JD-R model (Bakker and Demerouti, 2017) in this specific context, suggesting that in environments such as the ER, digital literacy may serve as a personal resource directly related to job demands (technological empowerment) while also showing indirect associations through its relationship with psychological resources (psychological empowerment). This finding extends the work of Boğa and Yilmaz (2024), who found that emergency department experience was associated with higher health literacy. It also responds to the call by Wilson (2024) for digital approaches to support nurses’ mental well-being. Consequently, for ER nurses, integrated approaches that combine digital skills training with psychological resilience cultivation may be particularly relevant for addressing work stress.

## 6 Implications

### 6.1 Theoretical implications

This study makes several theoretical contributions. First, it provides a refined integration of COR and JD-R theories by demonstrating that the efficacy of personal resources (digital literacy, resilience) is not universal but is profoundly shaped by the context of the work environment. This supports and extends the JD-R model’s emphasis on context by showing how different job demands (ER’s time pressure vs. ICU’s emotional toll) create distinct pathways for resource utilization. Second, the findings offer a nuanced application of COR theory. While COR posits that resources help prevent stress, our study shows that in extreme, resource-depleting environments like the ICU, even a key personal resource like resilience can have its protective function overwhelmed, highlighting the concept of resource caravans in the face of caravan passageways (Hobfoll, 1989). Finally, by identifying a dual-path mechanism specific to the ER, we provide a more complex picture of how digital literacy can function both as a direct job resource and as a resource caravan that builds psychological capital, deepening our understanding of technology’s role in occupational health.

### 6.2 Practical implications

Based on these findings, we propose differentiated intervention strategies tailored to departmental risk levels:

*Low-risk wards* (e.g., General wards): Focus on leveraging the mediating role of psychological resilience. Interventions may strengthen resilience through targeted programs, such as mindfulness training and peer support groups. Digital literacy training in these settings can be framed as a means to build confidence and self-efficacy, thereby enhancing resilience.

*Medium-risk wards* (e.g., Emergency room): Emphasize digital technology empowerment. This includes deploying intelligent decision-support systems, implementing digital literacy training focused on practical applications (e.g., optimizing EHR use, mobile support tools), and promoting micro-learning opportunities. Crucially, this should be combined with resilience training to activate the dual-path pattern identified in this study.

*High-risk wards* (e.g., ICU): Implement systemic solutions that extend beyond individual capacity. Given the observed attenuation of resilience’s protective effect, interventions focusing solely on individual resilience may be insufficient. This involves restructuring workflows, optimizing patient assignments, incorporating trauma-informed care and crisis simulation training, and providing robust, accessible psychological support to address the challenges of an environment where the protective association of resilience with work stress was not observed.

## 7 Conclusion

This study demonstrates that while digital literacy and psychological resilience are personal resources associated with lower work stress among nurses, their relationships with work stress are significantly and complexly moderated by the ward environment. A dual-path pattern (both direct and indirect) linking digital literacy to lower work stress was identified in the emergency room. In non-ICU departments, the association was observed only through the mediating role of psychological resilience. Conversely, the negative association between psychological resilience and work stress was significantly attenuated in the ICU setting. The ward environment itself emerged as a key contextual factor, independently accounting for a substantial portion of the variance in work stress.

## 8 Limitations and future research

Several limitations of this study should be acknowledged. First, the cross-sectional design, while revealing complex associative patterns among the variables and supporting the theoretical hypotheses, precludes definitive conclusions regarding causal direction or temporal sequence. For instance, although the mediation effect of psychological resilience is statistically supported, the proposed causal pathway (digital literacy → psychological resilience → work stress) requires validation through longitudinal studies or intervention experiments. Similarly, the moderating effects observed indicate differences in the strength of associations, but the underlying causal mechanisms warrant further exploration.

Second, the use of snowball sampling and digital-only data collection (via WeChat) may have introduced self-selection bias. Nurses who are more comfortable with digital tools or have higher digital literacy may have been more likely to participate, potentially influencing the observed associations. This digital recruitment method, combined with the sample being drawn exclusively from Western China, limits the generalizability of our findings to other geographical regions or healthcare contexts where digital infrastructure and literacy levels may differ. Additionally, all variables were measured using self-report data, which may introduce common method bias, although diagnostic tests suggested it was not a severe threat.

Future research should employ multi-wave longitudinal designs, incorporate objective measures of stress (e.g., physiological indicators), and utilize randomized controlled trials to verify the causal relationships and the efficacy of potential interventions. Investigating the long-term effects and dynamic interactions among department context, digital literacy, and psychological resilience on nurse work stress represents a critical direction for further inquiry.

## Data Availability

The original contributions presented in the study are included in the article/supplementary material, further inquiries can be directed to the corresponding author.

## Glossary

HWT: Hospital Ward Type
NWS: Nurses’ Work Stress
NDL: Nurses’ Digital Literacy
NPR: Nurses’ Psychological Resilience
ICU: Intensive Care Unit
ER: Emergency Room
GW: General Ward
SEM: Structural Equation Modeling
RMSEA: Root Mean Square Error of Approximation
GFI: Goodness-of-Fit Index
CFI: Comparative Fit Index
NFI: Normed Fit Index
IFI: Incremental Fit Index
TLI: Tucker-Lewis Index
PNFI: Parsimonious Normed Fit Index
PCFI: Parsimonious Comparative Fit Index
AIC: Akaike Information Criterion
VIF: Variance Inflation Factor
SR: Standardized Residual
SD: Standard Deviation
SE: Standard Estimate
